# Supplementary material for: Listeria monocytogenes Interferes with Host Cell Mitosis through Its Virulence Factors InlC and ActA
Source: Toxins (Basel). 2020 Jun 20;12(6):411. doi: 10.3390/toxins12060411 (PMC7354435; doi:10.3390/toxins12060411)
Supplement: Supplementary file 1 [file toxins-12-00411-s001.zip › toxins-820801 supplementary materials/toxins-820801 supplementary.docx]

Supplementary Materials: *Listeria monocytogenes* Interferes with Host Cell Mitosis through Its Virulence Factors InlC and ActA

Ana Catarina Costa, Jorge Pinheiro, Sandra A. Reis, Didier Cabanes and Sandra Sousa

**Table S1.** Cell cycle phases distribution (%) obtained upon DNA histograms quantification for three independent experiments.

| **Experiment** | **Phases** | **Asynch** | **NI 0h** | **Inf 0h** | **NI 2h** | **Inf 2h** |
| --- | --- | --- | --- | --- | --- | --- |
| #1 | G1/G0 | 54,4 | 13,9 | 14,9 | 42,5 | 36,0 |
|  | S | 19,4 | 26,5 | 26,4 | 24,7 | 25,6 |
|  | G2/M | 26,2 | 59,6 | 58,7 | 32,8 | 38,3 |
| #2 | G1/G0 | 54,0 | 5,8 | 7,5 | 44,2 | 34,3 |
|  | S | 31,6 | 20,1 | 23,4 | 19,8 | 22,8 |
|  | G2/M | 14,4 | 74,1 | 69,1 | 36,0 | 42,9 |
| #3 | G1/G0 | 43,4 | 3,1 | 3,8 | 32,8 | 41,4 |
|  | S | 37,4 | 12,2 | 9,7 | 10,9 | 16,2 |
|  | G2/M | 19,2 | 84,7 | 86,4 | 56,3 | 42,4 |


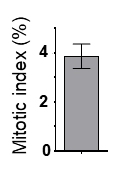


**Figure S1.** Ratio of mitotic cells in asynchronous Caco-2 cells. Data are mean ± SEM of three independent experiments.


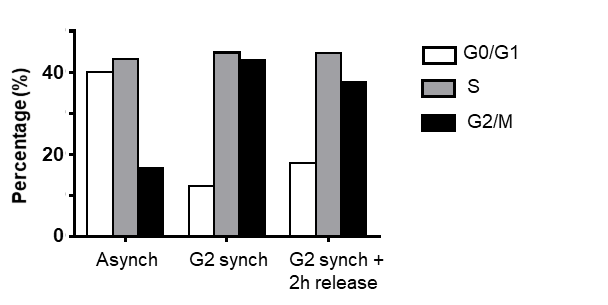


**Figure S2.** Cell cycle phase distribution in one of the performed experiments. Caco-2 cells were left asynchronous (Asynch), synchronized with RO-3306 (G2 synch), and released for 2 h after G2 synchronization (G2 synch + 2 h Release).


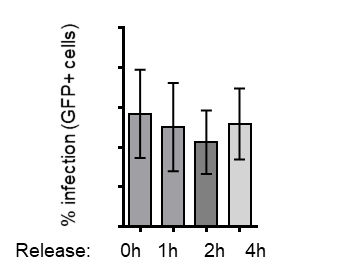


**Figure S3.** Rates of infection in Caco-2 cells 5 h post-infection. Cells were stopped before release or 1, 2 and 3 h after G2 synchronization with RO-3306 (20 h, 10 µ).


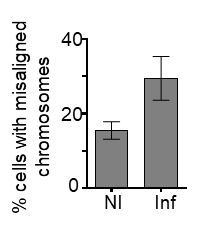


**Figure S4.** Percentage of mitotic cells with misaligned chromosomes in the BeWo cell line in non-infected (NI) and *Lm*-infected conditions (Inf).


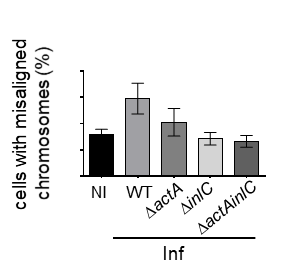


**Figure S5.** Percentage of BeWo mitotic cells with misaligned chromosomes in non-infected (NI) and *Lm*-infected conditions (Inf): with WT *Lm*, ∆*actA*, ∆*inlC* or ∆*actAinlC* deletion mutants.


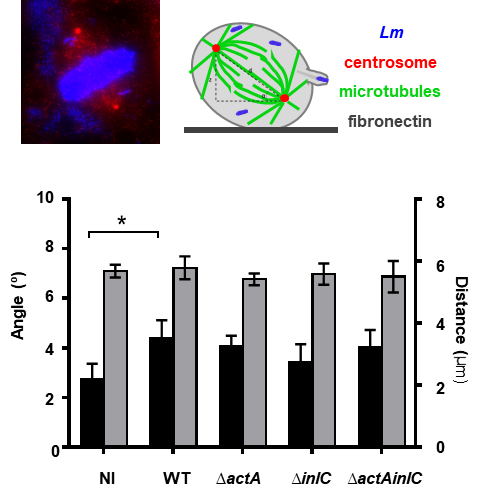


**Figure S6.** *Lm* infection does not dramatically interfere with mitotic spindle orientation and distance between the two centrosomes. Upper panel shows a microscopic Z-plane of a Caco-2 cell mitotic cell infectedby *Lm* and stained for gamma–tubulin (red) and DNA (blue). The scheme represents a *Lm*-infected mitotic cell: distance (d), angle (⍶) and Z-axis plane (z). The mitotic spindle parameters quantified in lower panel—spindle angle (black) and interpolar distance (grey)—were calculated according to the method depicted in the scheme. Data are means ± SEM of 4 independent experiments. * corresponds to *p* < 0.05.
